# High ratio of C-reactive protein to albumin is associated with hemorrhagic transformation and poor functional outcomes in acute ischemic stroke patients after thrombolysis

**DOI:** 10.3389/fnagi.2023.1109144

**Published:** 2023-02-16

**Authors:** Tong Xu, Lingfan Xia, Yucong Wu, Ye Xu, Xuan Xu, Wangyu Zhang, Congcong Zhou, Fangwang Fu, Yungang Cao, Zhao Han

**Affiliations:** Department of Neurology, The Second Affiliated Hospital and Yuying Children’s Hospital of Wenzhou Medical University, Wenzhou, China

**Keywords:** acute ischemic stroke, intravenous thrombolysis, recombinant tissue plasminogen activator, hemorrhagic transformation, functional outcome, inflammation, C-reactive protein/albumin ratio

## Abstract

**Background:**

In patients with acute ischemic stroke, hemorrhagic transformation (HT) is a common complication after intravenous thrombolysis (IVT). In this study, we evaluated the relationship between the ratio of C-reactive protein to albumin (CAR) before thrombolysis, HT, and functional outcomes in patients with acute ischemic stroke.

**Methods:**

We retrospectively analyzed data from 354 patients who received thrombolytic therapy at the Second Affiliated Hospital of the Wenzhou Medical University in China between July 2014 and May 2022. CAR was measured on admission, and HT was identified by cranial computed tomography (CT) within 24–36 h after treatment. Poor outcome was defined as a score on the modified Rankin Scale (mRS) > 2 at discharge. The multivariate logistic regression model was used to investigate the association between CAR, HT, and poor outcome after thrombolysis, respectively.

**Results:**

A total of 354 patients were analyzed, and their median CAR was 0.61 (interquartile range, 0.24–1.28). CAR was significantly higher in the 56 patients (15.8%) who experienced HT than in those who did not (0.94 vs. 0.56, *p* < 0.001), and the 131 patients (37.0%) who experienced poor outcome than in those who did not (0.87 vs. 0.43, *p* < 0.001). Multivariate logistic regression indicated that CAR was an independent risk factor for both HT and poor outcome. The risk of HT was significantly higher among patients whose CAR fell in the fourth quartile than among those with CAR in the first quartile (OR 6.64, 95% CI 1.83 to 24.17, *p* = 0.004). Patients with CAR in the third quartile were more likely to experience poor outcome (OR 3.35, 95% CI 1.32 to 8.51, *p* = 0.01), as were those in the fourth quartile (OR 7.33, 95% CI 2.62 to 20.50, *p* < 0.001), compared to patients with CAR in the first quartile.

**Conclusion:**

High ratio of C-reactive protein to albumin in individuals with ischemic stroke is associated with an increased risk of HT and poor functional outcomes after thrombolysis.

## Introduction

Intravenous thrombolysis (IVT) with recombinant tissue plasminogen activator (rt-PA) effectively improves functional outcomes in patients with acute ischemic stroke (AIS) (Emberson et al., 2014). However, thrombolytic therapy may cause hemorrhagic transformation (HT) (Yaghi et al., 2017), which can lead to early deterioration of neurological function, early death and poor prognosis (Mori et al., 2012; Yeo et al., 2013; Seners et al., 2015). Thus, a more objective and easily obtainable predictor is needed to identify high-risk patients of HT and conduct their individualized treatment.

Inflammation plays a pivotal role in vascular injury. Post-ischemic inflammatory responses that disrupt the blood–brain barrier may be responsible for hemorrhagic transformation, which then further worsens the prognosis of patients with acute ischemic stroke (Jickling et al., 2014). C-reactive protein, of which levels increase rapidly with inflammation or infection, has direct proinflammatory effects (Pasceri et al., 2000; Calabro et al., 2003) and has emerged as a strong independent risk factor for future cardiovascular events (Ridker, 2003). Serum albumin is a negative acute-phase protein, of which levels are linked to the intensity of the infection-triggered inflammatory response (Ritchie et al., 1999), and can directly protect vascular endothelium (Goldwasser and Feldman, 1997). Here we explored the combination of C-reactive protein and albumin, as an early serum biomarker, for predicting neurological outcomes in patients with acute ischemic stroke after thrombolysis. Indeed, the relationship between the ratio of C-reactive protein to albumin (CAR) and the prognosis of critically ill and cancer patients has been widely studied (Fairclough et al., 2009; Ranzani et al., 2013; Kinoshita et al., 2015), and CAR has already shown prognostic value in different types of cardiovascular diseases (Kinoshita et al., 2015; Yavuz et al., 2018; Mehmet and Kadriye, 2021), peripheral artery disease (Muhammed et al., 2022) and acute stroke (Jang et al., 2022).

The present study investigated whether the ratio of C-reactive protein to albumin might be associated with HT as well as poor functional outcomes in acute ischemic stroke patients after thrombolysis.

## Methods

### Patients

Our study retrospectively analyzed patients with acute ischemic stroke who received intravenous thrombolysis in the Second Affiliated Hospital of Wenzhou Medical University from July 2014 to May 2022. Any patient who received alteplase at 0.9 mg/kg until a maximum total dose of 90 mg within 4.5 h of stroke onset was included in the study unless (1) C-reactive protein and albumin levels at admission were unavailable, (2) computed tomography was not performed within 36 h after intravenous thrombolysis, (3) patients had an immune disorder or severe infection, defined as a history of cancer, hematologic disease, use of an immunosuppressant or the presence of sepsis on admission, (4) low-dose alteplase was used, or (5) thrombolysis was interrupted for reasons other than HT.

This study was approved by the Ethics Committee of the Second Affiliated Hospital and Yuying Children’s Hospital of Wenzhou Medical University. The Committee waived the requirement for written informed consent because patients or their legal guardians, at the time of treatment, consented for anonymized medical data to be analyzed and published for research purposes.

### Data collection

The following data were extracted from medical records: the demographic factors of age and sex; vascular risk factors, including hypertension, diabetes, hyperlipidemia, and current smoking; comorbidities such as atrial fibrillation, coronary artery disease, and stroke history (defined as previous ischemic stroke events); antithrombotic therapy before thrombolysis, blood pressure and stroke severity on admission, with severity assessed using the National Institutes of Health Stroke Scale (NIHSS) score. Blood test results also were collected, including levels of C-reactive protein, albumin, hemoglobin, fibrinogen, D-dimer, creatinine and glucose, white blood cell count and platelet count, international normalized ratio (INR), activated partial thromboplastin time (APTT); and cranial computed tomography (CT). Stroke was classified based on possible etiologies according to the TOAST classification (Adams et al., 1993).

Venous blood was analyzed using an automatic hematology analyzer (XE-5000, Sysmex, Kobe, Japan) and an automated biochemical analyzer (AU5800, Beckman Coulter, Brea, CA, United States). Blood was sampled before treatment in the emergency department. CAR was calculated by dividing C-reactive protein level (mg/L) by albumin level (g/dL), both of which were determined in the same blood sample.

### Assessments and outcomes

All patients underwent CT scanning once on admission and again within 24–36 h after intravenous thrombolysis. For the present study, CT images were retrospectively evaluated by two experienced neurologists who were blinded to the patient’s clinical data, and disagreements were settled by discussion. HT was defined as intracranial hemorrhage that was detected on the second but not first, CT. HT was subclassified as hemorrhagic infarction (HI) of type 1 or 2, or as parenchymal hemorrhage (PH) of type 1 or 2, based on European Cooperative Acute Stroke Study criteria (Hacke et al., 1995). In addition, we assessed functional neurological outcomes at discharge using the modified Rankin Scale (mRS). Poor outcome was defined as the absence of functional independence at discharge (modified Rankin Scale >2).

### Statistical analysis

Normally distributed continuous variables were expressed as mean ± SD, skewed continuous variables as median (interquartile range, IQR), and categorical variables as frequency (percentage). In univariate analysis, differences between continuous variables in two groups were assessed for significance using the t test or U-test as appropriate, while differences between categorical variables in two or more groups were assessed using Pearson’s chi-squared test or Fisher’s exact probabilities test as appropriate.

Unadjusted models and multivariate logistic regression were used to analyze the association of CAR with HT or poor outcome after thrombolysis. The model included CAR but not its individual components because of their cross-correlation. CAR was modeled either as a continuous variable, in which case odds ratios (ORs) were calculated per 1-SD increase; or in quartiles, in which case ORs were calculated relative to the first quartile. In multivariate logistic regression, model 1 was adjusted for age and sex, while model 2 was adjusted for age, sex and other variables that were associated with *p* < 0.1 in the univariate analysis.

We calculated areas under receiver operating characteristic curves (AUCs) to assess the ability of CAR to identify patients who experience HT or poor outcome. The optimal cut-off CAR was calculated by the Youden’s index, defined as the value that gave the greatest sum of specificity and sensitivity.

All statistical analyzes were performed using SPSS 26.0 for Windows (IBM, Armonk, NY, United States). Results associated with two-tailed *p* < 0.05 were considered statistically significant.

## Results

### Patient characteristics at baseline

**Figure 1:**
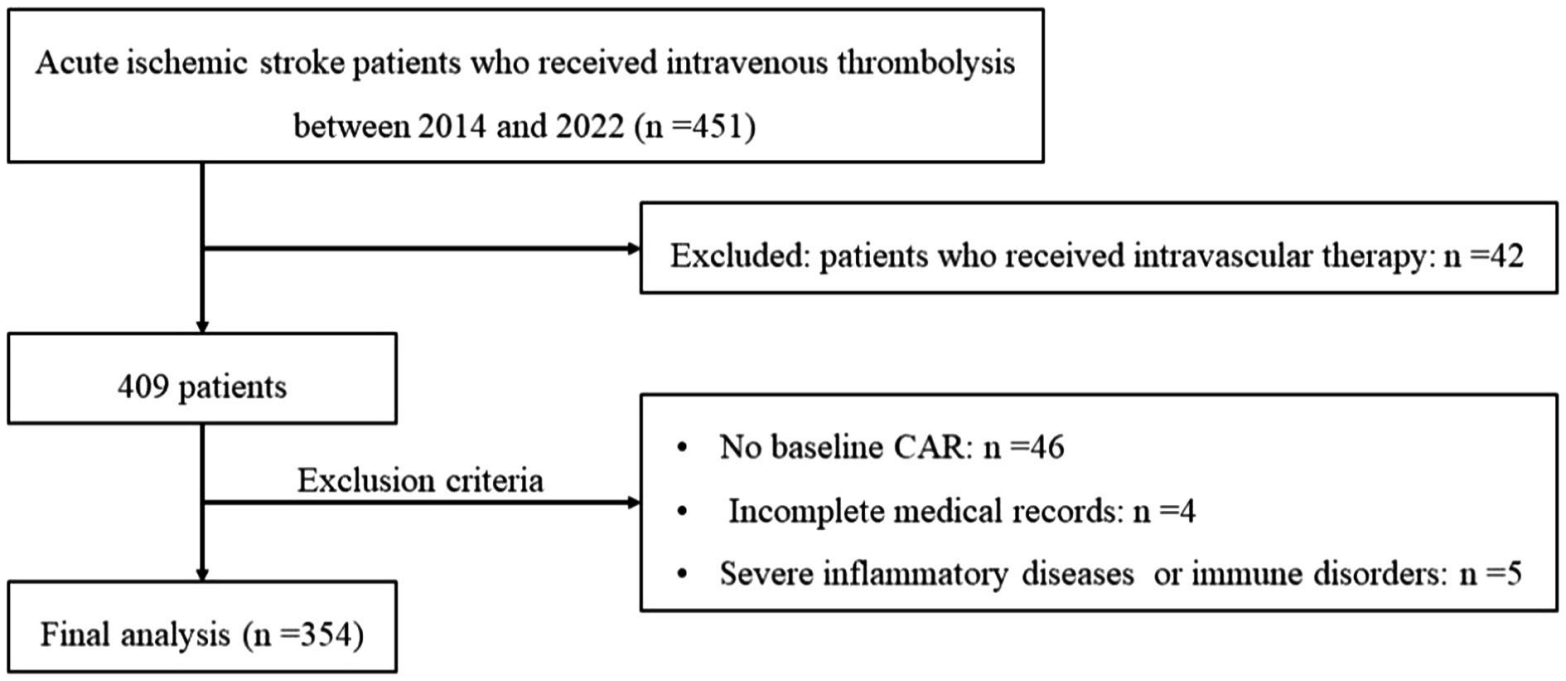
Flowchart of patient inclusion and analysis. CAR, ratio of C-reactive protein to albumin; HT, hemorrhagic transformation.

A total of 451 acute ischemic stroke patients who received intravenous thrombolysis treatment were screened for eligibility from July 2014 to May 2022. Based on the protocol (Figure 1), 97 patients were excluded, and 354 patients were included in the final analysis. The median age was 69 (range, 56 to 82 years), and 232 patients (65.5%) were men. The NIHSS score of the series on admission was 6 (range, 4 to 11) and the median mRS score at discharge was 2 (range, 1 to 3; Supplementary Table S1).

After thrombolysis, HT was observed in 56 patients (15.8%) and poor outcome was observed in 131 (37.0%) patients, respectively. HT was associated with a significantly higher incidence of atrial fibrillation, NIHSS score, diastolic blood pressure at admission, international normalized ratio, D-dimer level, and C-reactive protein level (*p* < 0.05; Table 1). Patients who experienced poor outcome were more likely to be older and female and to have a higher incidence of atrial fibrillation as well as diabetes mellitus, NIHSS score at admission, international normalized ratio, fibrinogen, D-dimer, hemoglobin and C-reactive protein level (*p* < 0.05; Table 2). When we compared patients in the high and low CAR groups, patients with CAR higher than the median value in the sample were older, had higher rates of atrial fibrillation, lower frequencies of small vessel occlusion stroke, and higher initial NIHSS and CRP levels than those with CAR lower than the median value (Supplementary Table S2).

**Table 1:** Comparison of characteristics between patients who experienced HT or not.

Characteristic	HT (*n* = 56)	No HT (*n* = 298)	*P*
**Demographics**			
Age, years	74 (63–83)	70 (61–79)	0.10
Male	32 (57.1)	200 (67.1)	0.15
**Vascular risk factors**			
Hypertension	44 (78.6)	228 (76.5)	0.74
Diabetes mellitus	18 (32.1)	93 (31.2)	0.89
Hyperlipidemia	20 (35.7)	141 (47.3)	0.11
Current smoking	8 (14.3)	94 (31.5)	0.009*
**Comorbidities**			
Atrial fibrillation	32 (57.1)	68 (22.8)	<0.001*
Coronary artery disease	6 (10.7)	23 (7.7)	0.43
Stroke history	8 (14.2)	47 (15.8)	0.78
**Medication history**			
Current antithrombotic therapy	15 (26.8)	51 (17.1)	0.09
**Clinical features**			
ONT, min	170 (127–213)	165 (114–198)	0.133
Initial NIHSS score	13 (7–18)	5 (3–9)	<0.001*
Baseline SBP, mmHg	158 (28)	158 (23)	1.00
Baseline DBP, mmHg	94 (19)	87 (16)	0.004*
**Laboratory tests**			
C-reactive protein, mg/L	4.17 (1.94–9.17)	2.31 (0.99–4.45)	<0.001*
Albumin, g/L	40.5 (38.8–43.8)	41.6 (39.6–44.0)	0.15
Ratio of C-reactive protein to albumin	0.94 (0.46–2.14)	0.56 (0.24–1.04)	<0.001*
Baseline glucose, mmol/L	7.4 (6.1–9.6)	7.0 (6.0–8.8)	0.26
White blood cells, 10^9^/l	7.2 (6.2–8.8)	7.1 (6.0–8.8)	0.69
Hemoglobin, g/dL	136 (125–149)	142 (130–152)	0.18
Platelets, 10^9^/l	190 (156–215)	194 (169–232)	0.09
Creatinine, μmol/L	73 (61–90)	73 (61–86)	0.59
INR	1.04 (1.00–1.11)	1.01 (0.96–1.06)	0.002*
APTT, s	33.4 (31.5–36.5)	34.4 (31.9–37.2)	0.43
Fibrinogen, g/L	3.33 (2.97–4.17)	3.22 (2.78–3.81)	0.12
D-Dimer, μg/ml	1.06 (0.52–1.70)	0.46 (0.29–1.02)	<0.001*
**Stroke subtype**			<0.001*
Large-artery atherosclerosis	18 (32.1)	115 (38.6)	
Small-vessel occlusion	4 (7.1)	77 (25.8)	
Cardioembolic	28 (50.0)	67 (22.5)	
Other determined etiology	4 (7.1)	5 (1.7)	
Undetermined etiology	2 (3.6)	34 (11.4)	

Values are *n* (%), median (IQR), or mean ± SD, unless otherwise noted. APTT, activated partial thromboplastin time; DBP, diastolic blood pressure; HT, hemorrhagic transformation; INR, international normalized ratio; MAC, middle cerebral artery; NIHSS, National Institutes of Health Stroke Scale; ONT, onset-to-treatment time; SBP, systolic blood pressure. *p < 0.05.

**Table 2:** Comparison of characteristics between patients who experienced poor outcome or not.

Characteristic	No poor outcome (*n* = 223)	Poor outcome (*n* = 131)	*P*
**Demographics**
Age, years	69 (60–78)	74 (64–82)	0.002*
Male	162 (72.6)	70 (53.4)	<0.001*
**Vascular risk factors**			
Hypertension	170 (76.2)	102 (77.9)	0.73
Diabetes mellitus	61 (27.4)	50 (38.2)	0.03*
Hyperlipidemia	103 (46.2)	58 (44.3)	0.73
Current smoking	75 (33.6)	27 (20.6)	0.009*
**Comorbidities**			
Atrial fibrillation	47 (21.1)	53 (40.5)	<0.001*
Coronary artery disease	18 (8.1)	11 (8.4)	0.91
Stroke history	35 (15.7)	20 (15.3)	0.92
**Medication history**			
Current antithrombotic therapy	39 (17.5)	27 (20.6)	0.47
**Clinical features**			
ONT, min	162 (111–195)	172 (129–215)	0.12
NIHSS score	5 (3–8)	11 (6–16)	<0.001*
Baseline SBP, mmHg	153 (23)	160 (25)	0.25
Baseline DBP, mmHg	87 (16)	90 (18)	0.07
**Laboratory test**			
C-reactive protein, mg/L	1.81 (0.82–3.82)	3.44 (2.07–9.00)	<0.001*
Albumin, g/L	41.5 (39.9–44.1)	41.2 (38.9–43.8)	0.29
Ratio of C-reactive protein to albumin	0.43 (0.19–0.89)	0.87 (0.49–2.15)	<0.001*
Baseline Glucose, mmol/L	6.9 (5.9–8.6)	7.5 (6.1–9.6)	0.05
White Blood Cells, 10^9^/l	7.0 (6.0–8.6)	7.3 (6.1–9.2)	0.19
Hemoglobin, g/dL	142 (133–154)	137 (125–149)	0.03*
Platelet, 10^9^/l	194 (167–228)	190 (165–233)	0.87
Creatinine, umol/L	75 (63–87)	70 (60–86)	0.11
INR	1.00 (0.96–1.05)	1.03 (0.97–1.11)	0.004*
APTT, s	34.5 (32.1–37.1)	33.9 (31.2–37.3)	0.42
Fibrinogen, g/L	3.16 (2.75–3.72)	3.36 (2.90–4.21)	0.003*
D-Dimer, ug/ml	0.44 (0.28–0.92)	0.76 (0.42–1.39)	<0.001*
**Stroke subtype**			<0.001*
Large artery atherosclerosis	87 (39)	46 (35.1)	
Small vessel occlusion	63 (28.3)	18 (13.7)	
Cardioembolic	42 (18.8)	53 (40.5)	
Other determined etiology	3 (1.3)	6 (4.6)	
Undetermined etiology	28 (12.6)	8 (6.1)	

Values are *n* (%), median (IQR), or mean ± SD, unless otherwise noted. APTT, activated partial thromboplastin time; DBP, diastolic blood pressure; HT, hemorrhagic transformation; INR, international normalized ratio; MAC, middle cerebral artery; NIHSS, National Institutes of Health Stroke Scale; ONT, onset-to-treatment time; SBP, systolic blood pressure. *p < 0.05.

### Relationship between CAR and the risk of HT

**Figure 2:**
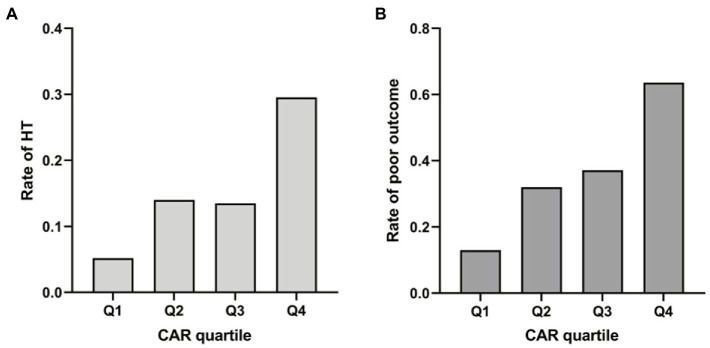
**(A)** Rate of hemorrhagic transformation (HT) by CAR quartile. **(B)** Rate of poor outcome by CAR quartile.

Median CAR was 0.61 (0.24–1.28) across all patients, and HT was significantly associated with higher CAR (0.94 vs. 0.56, *p* < 0.001). Patients in the highest quartile had a significantly higher incidence of HT than those in the lowest quartile (Figure 2A; Supplementary Table S4). In the multivariable logistic regression analysis, CAR was identified as an independent risk factor for HT after being adjusted for confounders. The result was found when CAR was treated as a quartile variable [OR 6.64 for the fourth quartile (> 1.28) relative to the first quartile (< 0.24), 95% CI 1.83–20.17, *p* = 0.004; Table 3].

**Table 3:** Multivariate logistic regression to assess the potential relationship between CAR and HT.

	Unadjusted model		Model 1		Model 2	
	OR (95%CI)	*P*	OR (95%CI)	*P*	OR (95%CI)	*P*
**CAR as continuous variable**						
Per 1-SD increase	1.56 (0.84–2.79)	0.13	1.50 (0.84–2.68)	0.18	1.70 (0.86–3.36)	0.13
**CAR in quartiles**						
Q1 (<0.24)	Reference		Reference		Reference	
Q2 (0.24–0.61)	2.97 (0.94–9.42)	0.06	2.84 (0.89–9.06)	0.08	2.72 (0.74–9.94)	0.13
Q3 (0.61–1.28)	2.84 (0.88–9.22)	0.08	2.76 (0.85–9.00)	0.09	2.60 (0.71–9.48)	0.15
Q4 (>1.28)	7.65 (2.53–23.12)	<0.001*	7.19 (2.34–22.06)	0.001*	6.64 (1.83–24.17)	0.004*

Model 1: adjusted for age and sex. Model 2: same as in Model 1 as well as atrial fibrillation, current smoking, ongoing antithrombotic therapy, baseline DBP, NIHSS score, platelet, D-Dimer, INR and stroke subtype. CI, confidence interval; OR, odds ratio. *p < 0.05.

### Relationship between CAR and clinical outcome

CAR in patients who experienced poor outcome was significantly higher than those who did not (0.87 vs. 0.43, *p* < 0.001). Patients were then stratified according to whether they suffered HT or not. Subgroup analysis also showed a positive association between CAR and poor outcome risk among those who experienced HT (1.44 vs. 0.68, *p* = 0.04; Supplementary Table S3). Rates of poor outcome increased by CAR quartiles (Figure 2B; Supplementary Table S4). Univariate logistic regression analysis showed that CAR had an independent positive relationship with the poor outcome when CAR was treated as a continuous variable. This finding stabilized in model 1 after adjusting for age and sex (OR 3.13 per 1-SD increase, 95% CI 1.13–8.69, *p* = 0.03; Table 4). When treated as a quartile variable, CAR remained an independent predictor for poor outcome [OR 3.35 for the third quartile (0.61–1.28) relative to the first quartile, 95% CI 1.32–8.51, *p* = 0.01; OR 7.33 for fourth quartile (> 1.28) relative to the first quartile, 95% CI 2.62–20.50, *p* < 0.001; Table 4].

**Table 4:** Multivariate logistic regression to assess the potential relationship between CAR and poor outcome.

	Unadjusted model		Model 1		Model 2	
	OR (95%CI)	*P*	OR (95%CI)	*P*	OR (95%CI)	*P*
**CAR as continuous variable**						
Per 1-SD increase	3.96 (1.36–1.49)	0.01*	3.13 (1.13–8.69)	0.03*	1.14 (0.53–2.44)	0.74
**CAR in quartiles**						
Q1 (<0.24)	Reference		Reference		Reference	
Q2 (0.24–0.61)	3.15 (1.44–6.92)	0.04*	2.94 (1.32–6.53)	0.08*	2.50 (1.01–6.24)	0.05
Q3 (0.61–1.28)	3.95 (1.79–8.71)	0.01*	3.85 (1.72–8.62)	0.01*	3.35 (1.32–8.51)	0.01*
Q4 (>1.28)	11.73 (5.30–25.93)	<0.001*	11.08 (4.91–25.04)	<0.001*	7.33 (2.62–20.50)	<0.001*

Model 1: adjusted for age and sex. Model 2: same as in Model 1 as well as arial fibrillation, diabetes mellitus, current smoking, NIHSS score, baseline DBP, baseline glucose, hemoglobin, fibrinogen, D-Dimer, INR and stroke subtype. CI, confidence interval; OR, odds ratio. *p < 0.05.

In addition, receiver operating characteristic curve (ROC) analysis indicated a significant association between the risk of HT and a CAR cut-off value of >1.38, with a sensitivity of 44.6%, specificity of 81.5%, and AUC of 65.8% (95% CI 58.0–73.6%, *p* < 0.001). At the optimal cut-off of 0.42, CAR was able to differentiate between patients in our study who experienced poor outcome or not with a sensitivity of 82.4%, specificity of 49.8%, and AUC of 70.9% (95% CI 65.4–76.5%, *p* < 0.001).

## Discussion

To our knowledge, this is one of the few studies to investigate an association between CAR before thrombolysis and post-thrombolysis early neurological outcomes. In this study, we found that higher CAR was associated with a greater risk of HT and worse prognosis in patients with acute ischemic stroke after thrombolysis, which may help to identify high-risk patients and conduct their individualized treatment.

Our study identifies CAR as an independent factor for predicting prognosis in acute ischemic stroke patients after thrombolysis and explores the possibility of CAR application in various clinical diseases. CAR has been proven to be a predictor of infection burdens in many infectious diseases (Ranzani et al., 2013; Oh et al., 2018; Bai et al., 2019). Previous studies have shown that elevated CAR is related to major adverse cardiac events or cardiovascular mortality among patients with coronary artery disease (Yavuz et al., 2018; Mehmet and Kadriye, 2021; Sabanoglu and Inanc, 2022) as well as all-cause mortality among patients with peripheral artery disease (Muhammed et al., 2022). Several recent studies also provided compelling evidence that early CAR determination may help to predict the in-hospital mortality of patients with spontaneous intracerebral hemorrhage (Bender et al., 2020) and patients with acute stroke (Jang et al., 2022).

The present study confirms that CAR can be served as a useful indicator for reflecting systemic inflammatory state and predicting the prognosis among acute stroke patients (Bender et al., 2020; Jang et al., 2022). The effect of inflammation on the pathogenesis and progression of ischemic stroke has been more clearly understood in recent years (Anrather and Iadecola, 2016; Becker and Buckwalter, 2016; Chamorro et al., 2016; Shichita et al., 2017; Rust et al., 2018; Shi et al., 2019), and post-ischemic inflammatory responses can cause hemorrhagic transformation and neurological deterioration by inducing oxidative stress that further disrupt the blood–brain barrier (Jickling et al., 2014; Arba et al., 2020). The new parameter, CAR reflects the stability of albumin and C-reactive protein levels inside the body, which is thoughted to be a better indicator of the inflammatory status compared to C-reactive protein or albumin separately (Fairclough et al., 2009; Ranzani et al., 2013; Kinoshita et al., 2015). C-reactive protein, an important indicator of increased inflammation, of which elevation is common in patients with acute ischemic stroke, is produced by hepatocytes and regulated by proinflammatory cytokines (Iwata et al., 2006). During the onset of the inflammatory response, C-reactive protein promotes the release of tissue factor from peripheral blood monocytes as well as endothelial cells (Cermak et al., 1993; Napoleone et al., 2002) and induces the expression and activity of plasminogen activator inhibitor-1 in endothelial cells (Devaraj et al., 2003), which may contribute to the progression of cerebral tissue injury (Vila et al., 2000). It has been established that C-reactive protein levels can reflect the extent of cerebral infarction (Di Napoli et al., 2001), and elevated levels of C-reactive protein are associated with increased in-hospital mortality as well as long-term poor functional outcomes in patients with ischemic stroke (Lee et al., 2020). However, during persistent systemic inflammation, the production of albumin and prealbumin was inhibited (Heinrich et al., 1990; Reuben et al., 2002), indicating that lower levels of serum albumin may be associated with cytokine activation and ongoing inflammation (Ritchie et al., 1999). Serum albumin can also directly protect vascular endothelium. Higher albumin levels may exert antioxidant effects by triggering vasodilation, binding toxins, preventing platelets from aggregation, and protecting endothelial cells against apoptotic death thereby protecting the vascular endothelium from damage (Goldwasser and Feldman, 1997).

Another possible explanation is that the severity of index stroke could be a connector between CAR, HT, and poor functional outcomes. According to our research, the group of patients with CAR higher than the median value showed a higher initial NIHSS score and a decreased number of small vessel occlusion strokes. It is well known that stroke severity and large stroke volumes are strong predictors of HT and poor functional outcomes (Mori et al., 2012; Emberson et al., 2014; Seners et al., 2015). Therefore, the CAR may serve as a marker for patients with severe stroke who are vulnerable to HT and poor functional outcomes.

Similar to our findings, another sample of Asian patients observed that higher CAR was significantly associated with worse functional outcomes in patients with acute stroke (Jang et al., 2022). Differently, the populations they studied were patients with acute stroke including cerebral infarction, intracranial hemorrhage, and subarachnoid hemorrhage, while patients who received intravenous thrombolysis were not studied separately. Additionally, the relationship between CAR and the risk of HT was not analyzed in their study. We also did not find any relation between CAR and in-hospital mortality after thrombolysis, which could be explained by the small sample size and low clinical severity of ischemic stroke at admission. Future work is needed for a larger sample size to clarify the effects of CAR on mortality in patients with acute ischemic stroke after thrombolysis.

In the current study, HT was observed in 15.8% of our patients, which is consistent with the range of 10–43% reported in previous studies (Yaghi et al., 2017). As in other studies, we found that atrial fibrillation and higher NIHSS score were significantly associated with increased risk of HT and worse functional outcomes, confirming results from studies of reperfusion therapy (Yaghi et al., 2017).

Our findings should be interpreted with caution given some limitations. Our sample came from a single center, and we measured CAR only once during hospitalization, yet it can vary during treatment. The fact that we consecutively recruited patients treated by tissue plasminogen activator without restrictive inclusion or exclusion criteria implies that our findings can be extended to all stroke subtypes, but this should be confirmed in future work. Last, the effects of vascular obstruction sites and patients which received endovascular treatment should be considered before generalizing our results to the clinical field.

## Conclusion

Our study identifies CAR as an independent predictor of HT and poor functional outcomes among acute ischemic stroke patients after intravenous thrombolysis. Thus, CAR may be an objective and easily obtainable predictor to assist in identifying at-risk patients as well as optimizing treatment. CAR may also be a useful inclusion criterion in clinical trials involving thrombolysis. Our findings justify further investigation into the complex relationships between inflammation and HT and other complications.

## Data availability statement

The raw data supporting the conclusions of this article will be made available by the authors, without undue reservation.

## Ethics statement

The studies involving human participants were reviewed and approved by the Department of Neurology at The Second Affiliated Hospital and Yuying Children’s Hospital of Wenzhou Medical University, Wenzhou, China. Due to the retrospective nature of the study, the requirement for written informed consent was waived by the Second Affiliated Hospital of Wenzhou Medical University.

## Author contributions

TX wrote the first draft of the article. All author contributed to the proposal and design of the study as well as the collection, analysis and interpretation of the data. All authors contributed to the article and approved the submitted version.

## Funding

This study was funded by the National Natural Science Foundation of China (82271344) and Clinical Scientific Research Foundation of the Second Affiliated Hospital of Wenzhou Medical University (SAHoWMU-CR2017-01-212).

## Conflict of interest

The authors declare that the research was conducted in the absence of any commercial or financial relationships that could be construed as a potential conflict of interest.

## Publisher’s note

All claims expressed in this article are solely those of the authors and do not necessarily represent those of their affiliated organizations, or those of the publisher, the editors and the reviewers. Any product that may be evaluated in this article, or claim that may be made by its manufacturer, is not guaranteed or endorsed by the publisher.

## Supplementary material

The Supplementary material for this article can be found online at: https://www.frontiersin.org/articles/10.3389/fnagi.2023.1109144/full#supplementary-material
