# Supplementary material for: High ratio of C-reactive protein to albumin is associated with hemorrhagic transformation and poor functional outcomes in acute ischemic stroke patients after thrombolysis
Source: Front Aging Neurosci. 2023 Feb 16;15:1109144. doi: 10.3389/fnagi.2023.1109144 (PMC9978514; doi:10.3389/fnagi.2023.1109144)
Supplement: Supplementary file 1 [file Table_1.docx]

Table S1. Main characteristics of the study population (n = 354)

| **Parameter** | **Value** |
| --- | --- |
| ***Demographics*** |  |
| Age, years | 69 (56-82) |
| Male | 232 (65.5) |
| ***Vascular risk factors*** |  |
| Hypertension | 272 (76.8) |
| Diabetes mellitus | 111 (31.4) |
| Hyperlipidemia | 161 (45.5) |
| Current smoking | 102 (28.8) |
| ***Comorbidities*** |  |
| Atrial fibrillation | 100 (28.2) |
| Coronary artery disease | 29 (8.2) |
| Stroke history | 55 (15.5) |
| ***Medication history*** |  |
| Current antithrombotic therapy | 59 (16.7) |
| ***Clinical features*** |  |
| ONT, min | 170 (125–210) |
| Initial NIHSS score | 6 (4-11) |
| Baseline SBP, mmHg | 158±24 |
| Baseline DBP, mmHg | 88±17 |
| ***Laboratory test*** |  |
| C-reactive protein, mg/L | 2.55 (1.14-5.33) |
| Albumin, g/L | 41.7±3.6 |
| Ratio of C-reactive protein to albumin | 0.61 (0.24-1.28) |
| Baseline glucose, mmol/L | 7.1 (6.0-8.9) |
| White blood cell count, 10^9^ /L | 7.1 (6.0-8.8) |
| Hemoglobin, g/dL | 140.1±18.7 |
| Platelet count, 10^9^ /L | 193 (167-228) |
| Creatinine, μmol/L | 73 (61-86) |
| INR | 1.03±0.11 |
| APTT, s | 34.7±4.6 |
| Fibrinogen, g/L | 3.26 (2.80-3.95) |
| D-Dimer, ug/ml | 0.53 (0.30-1.06) |
| ***Stroke subtype*** |  |
| Large artery atherosclerosis | 133 (37.6) |
| Small vessel occlusion | 81 (22.9) |
| Cardioembolic | 95 (26.8) |
| Other determined etiology | 9 (2.5) |
| Undetermined etiology | 36 (10.2) |
| ***mRS score at discharge*** | 2 (1-3) |
| 0 | 32 (9.0) |
| 1 | 127 (35.9) |
| 2 | 64 (18.1) |
| 3 | 46 (13.0) |
| 4 | 49 (13.8) |
| 5 | 34 (9.6) |
| 6 | 2 (0.6) |

Values are n (%), median (interquartile range) or mean ± SD, unless otherwise noted.

APTT, activated partial thromboplastin time; CAR, ratio of C-reactive protein to albumin; DBP, diastolic blood pressure; HT, hemorrhagic transformation; INR, international normalized ratio; mRS, modified Rankin scale; NIHSS, National Institutes of Health Stroke Scale; ONT, onset-to-treatment time; SBP, systolic blood pressure.

* P < 0.05.

Table S2 Baseline characteristics of patients with low and high CARs

| **Characteristic** | **Low CAR，**  **(<0.61)** | **High CAR,**  **(≥0.61)** | **P** |
| --- | --- | --- | --- |
| ***Demographics*** |  |  |  |
| Age, years | 68 (60-77) | 75 (64-82) | 0.001* |
| Male | 118 (66.7) | 114 (64.4) | 0.66 |
| ***Vascular risk factors*** | | | |
| Hypertension | 133 (75.1) | 139 (78.5) | 0.45 |
| Diabetes mellitus | 50 (28.2) | 61 (34.5) | 0.21 |
| Hyperlipidemia | 82 (46.3) | 79 (44.6) | 0.75 |
| Current Smoking | 54 (30.5) | 48 (27.1) | 0.48 |
| ***Comorbidities*** | | | |
| Atrial fibrillation | 37 (20.9) | 63 (35.6) | 0.002* |
| Coronary artery disease | 11 (6.2) | 18 (10.2) | 0.18 |
| Stroke history | 31 (17.5) | 24 (13.6) | 0.30 |
| ***Medication history*** |  |  |  |
| Current antithrombotic therapy | 29 (16.4) | 37 (20.9) | 0.28 |
| ***Clinical features*** |  |  |  |
| Initial NIHSS score | 6 (3-9) | 7 (4-13) | 0.006* |
| Baseline SBP, mmHg | 158 (143-172) | 157 (140-174) | 0.72 |
| Baseline DBP, mmHg | 87 (77-97) | 85 (75-99) | 0.77 |
| ***Laboratory test*** | | | |
| C-reactive protein, mg/L | 1.14 (0.66-1.74) | 5.31 (3.44-8.89) | <0.001* |
| Albumin, g/L | 41.9 (40.1-44.3) | 40.9 (38.9-43.7) | 0.008* |
| Baseline glucose, mmol/L | 7.0 (5.8-8.8) | 7.1 (6.1-9.1) | 0.39 |
| White blood cells count, 10^9^ /L | 6.7 (5.9-8.4) | 7.5 (6.3-9.3) | 0.001* |
| Hemoglobin, g/dL | 142 (132-153) | 140 (126-152) | 0.12 |
| Platelet count, 10^9^ /L | 191 (168-226) | 195 (163-233) | 0.54 |
| Creatinine, μmol/L | 74 (61-87) | 73 (61-87) | 0.80 |
| INR | 1.00 (0.95-1.05) | 1.03 (0.98-1.10) | <0.001* |
| APTT, s | 33.8 (31.7-36.5) | 35.0 (31.9-37.6) | 0.07 |
| Fibrinogen, g/L | 2.97 (2.65-3.41) | 3.59 (3.09-4.29) | <0.001* |
| D-Dimer, μg/ml | 0.47 (0.27-1.06) | 0.59 (0.34-1.21) | 0.006* |
| ***Stroke subtype*** |  |  | 0.007* |
| Large artery atherosclerosis | 68 (38.4) | 65 (36.7) | 0.74 |
| Small vessel occlusion | 53 (29.9) | 28 (15.8) | 0.002* |
| Cardioembolic | 36 (20.3) | 59 (33.3) | 0.006* |
| Other determined etiology | 4 (2.3) | 5 (2.8) | 1.00 |
| Undetermined etiology | 16 (9.0) | 20 (11.3) | 0.48 |
| HT | 18 (10.2) | 38 (21.5) | 0.004* |
| sHT | 5 (2.8) | 9 (5.1) | 0.28 |
| PH | 9 (5.1) | 13 (7.3) | 0.38 |
| ***mRS score at discharge*** | 1 (1-2) | 3 (1-4) | <0.001* |
| 0 | 26 (14.7) | 6 (3.4) |  |
| 1 | 77 (43.5) | 50 (28.2) |  |
| 2 | 32 (18.1) | 32 (18.1) |  |
| 3 | 21 (11.9) | 25 (14.1) |  |
| 4 | 14 (7.9) | 35 (19.8) |  |
| 5 | 7 (4.0) | 27 (15.3) |  |
| 6 | 0 (0) | 2 (1.1) |  |

Values are n (%), median (interquartile range) or mean ± SD, unless otherwise noted.

APTT, activated partial thromboplastin time; CAR, ratio of C-reactive protein to albumin; DBP, diastolic blood pressure; HT, hemorrhagic transformation; INR, international normalized ratio; MAC, middle cerebral artery; mRS, modified Rankin scale; NIHSS, National Institutes of Health Stroke Scale; PH, parenchymal hemorrhage; SBP, systolic blood pressure; sHT, symptomatic hemorrhagic transformation.

* P < 0.05.

Table S3 Comparison of characteristics between HT patients who experienced poor outcome or not.

| **Characteristic** | **No poor outcome (n=14)** | **Poor outcome (n=42)** | **P** |
| --- | --- | --- | --- |
| ***Demographics*** |  |  |  |
| Age, years | 72 (61-83) | 74 (63-82) | 0.87 |
| Male | 10 (71.4) | 22 (52.4) | 0.21 |
| ***Vascular risk factors*** | | | |
| Hypertension | 12 (85.7) | 32 (76.2) | 0.71 |
| Diabetes mellitus | 3 (21.4) | 15 (35.7) | 0.51 |
| Hyperlipidemia | 4 (28.6) | 16 (38.1) | 0.52 |
| Current smoking | 2 (14.3) | 6 (14.3) | 1.00 |
| ***Comorbidities*** | | | |
| Atrial fibrillation | 6 (42.9) | 26 (61.9) | 0.21 |
| Coronary artery disease | 2 (14.3) | 4 (9.5) | 0.63 |
| Stroke history | 2 (14.3) | 6 (14.3) | 1.00 |
| ***Medication history*** |  |  |  |
| Current antithrombotic therapy | 2 (14.3) | 13 (31.0) | 0.38 |
| ***Clinical features*** |  |  |  |
| ONT, min | 168 (113–199) | 170 (125–213) | 0.35 |
| Initial NIHSS score | 8 (5-12) | 14 (8-19) | 0.02* |
| Baseline SBP, mmHg | 152±27 | 160±28 | 0.39 |
| Baseline DBP, mmHg | 83 (78-89) | 96 (82-106) | 0.02* |
| ***Laboratory tests*** | | | |
| C-Reactive Protein, mg/L | 2.70 (1.38-4.91) | 5.49 (2.04-12.36) | 0.05 |
| Albumin, g/L | 40.4 (39.1-41.4) | 40.7 (38.8-44.1) | 0.58 |
| Ratio of C-reactive protein to albumin | 0.68 (0.35-1.13) | 1.44 (0.49-2.89) | 0.04* |
| Baseline glucose, mmol/L | 6.7 (5.8-8.8) | 7.6 (6.1-10.1) | 0.28 |
| White blood cells, 10^9^ /L | 7.1 (6.4-8.0) | 7.3 (6.2-9.2) | 0.87 |
| Hemoglobin, g/dL | 134±15 | 140±23 | 0.34 |
| Platelets, 10^9^ /L | 199 (174-223) | 188 (152-213) | 0.30 |
| Creatinine, mmol/L | 82 (58-115) | 71 (61-87) | 0.32 |
| INR | 1.01 (0.99-1.04) | 1.06 (1.00-1.12) | 0.06 |
| APTT, s | 34.4 (32.4-36.1) | 33.2 (31.5-37.6) | 0.73 |
| Fibrinogen, g/L | 3.58 (3.00-4.16) | 3.29 (2.83-4.20) | 0.61 |
| D-Dimer, mg/ml | 0.54 (0.43-1.70) | 1.14 (0.57-1.87) | 0.14 |
| ***Stroke subtype*** |  |  | 0.73 |
| Large-artery atherosclerosis | 6 (42.9) | 12 (28.6) |  |
| Small-vessel occlusion | 1 (7.1) | 3 (7.1) |  |
| Cardioembolic | 5 (35.7) | 23 (54.8) |  |
| Other determined etiology | 1 (7.1) | 3 (7.1) |  |
| Undetermined etiology | 1 (7.1) | 1 (2.4) |  |

Values are n (%), median (IQR), or mean ± SD, unless otherwise noted.

APTT, activated partial thromboplastin time; DBP, diastolic blood pressure; HT, hemorrhagic transformation; INR, international normalized ratio; MAC, middle cerebral artery; NIHSS, National Institutes of Health Stroke Scale; ONT, onset-to-treatment time; SBP, systolic blood pressure. * P < 0.05.

Table S4 Clinical outcomes of acute ischemic stroke patients after thrombolysis with CAR quartiles.

| **CAR in quartiles** | Q1 (<0.24) | Q2 (0.24-0.61) | Q3 (0.61-1.28) | Q4 (＞1.28) | P |
| --- | --- | --- | --- | --- | --- |
| HT | 4 (5.2) | 14 (14.0) | 12 (13.5) | 26 (29.5) ^c^ | <0.001* |
| Poor outcome | 10 (13.0) | 32 (32.0) ^a^ | 33 (37.1) ^b^ | 56 (63.6) ^c, d, e^ | <0.001* |

Values are n (%), median (interquartile range) or mean ± SD, unless otherwise noted.

CAR, ratio of C-reactive protein to albumin; HT, hemorrhagic transformation.

^a^ This variable is different significantly between Q1 and Q2, P _adjusted_ < 0.05

^b^ This variable is different significantly between Q1 and Q3, P _adjusted_ < 0.05

^c^ This variable is different significantly between Q1 and Q4, P _adjusted_ < 0.05

^d^ This variable is different significantly between Q2 and Q4, P _adjusted_ < 0.05

^e^ This variable is different significantly between Q3 and Q4, P _adjusted_ < 0.05

* P < 0.05.
